# Gut virome characteristics associated with early onset of anemia and neurodevelopmental delay in preterm infants

**DOI:** 10.1016/j.isci.2025.113578

**Published:** 2025-09-16

**Authors:** Shuqiang Ren, Du Zhang, Xingwei Shi, Tianze Li, Qi Hu, Yance Feng, Chenghao Hu, Siting Feng, Yanna Zhu, Fei Gao

**Affiliations:** 1 Genome Analysis Laboratory of the Ministry of Agriculture and Rural Affairs, Agricultural Genomics Institute at Shenzhen, Chinese Academy of Agricultural Sciences, Shenzhen, China; 2 Department of Medical Genetics, The Second Xiangya Hospital of Central South University, Changsha, China; 3 Department of Geriatric Medicine, South China Hospital, Medical School, Shenzhen University, Shenzhen, China; 4 Department of Maternal and Child Health, School of Public Health, Sun Yat-sen University, Guangzhou, China; 5 E-Gene Co. Ltd, Shenzhen, China; 6 Comparative Pediatrics and Nutrition, Department of Veterinary and Animal Sciences, Faculty of Health and Medical Sciences, University of Copenhagen, Copenhagen, Denmark

**Keywords:** Hematology, Pediatrics, Microbiome

## Abstract

Early-onset anemia (EOA) and neurodevelopmental delay (NDD) are highly prevalent in preterm infants, causing substantial long-term health impacts. This study aimed to identify distinctive gut virome characteristics and their associations with EOA and NDD. We hypothesized that gut microbial colonization types and bacteriophage profiles may be risk factors for NDD in preterm infants with EOA. Fecal samples from 107 healthy preterm infants within the first week of life underwent virome and 16S rRNA sequencing. Consensus clustering of viral species signatures divided infants into four groups. The high EOA risk group showed significantly higher virome alpha diversity. Enriched *Circoviridae* sp. and *uncultured Caudoviridae phage*, along with reduced *CRESS virus* sp., were linked to elevated NDD risk. *Geobacillus virus Tp84*—the only bacteriophage exhibiting both temperate and virulent lifestyles—was associated with high EOA risk but low NDD risk. These findings highlight the role of gut virome in EOA and NDD pathogenesis, suggesting potential for targeted bacteriophage-based interventions to mitigate EOA-related NDD in preterm infants.

## Highlights

• Gut virome diversity is elevated in preterm infants who are at high risk of earlyonset anemia (EOA)
• Specific viral taxa are associated with an increased risk of neurodevelopmental delay (NDD)
• *Tp84* phage associates with higher EOA but lower NDD risk, suggesting context-dependent effects

## Graphical abstract

**Figure:**
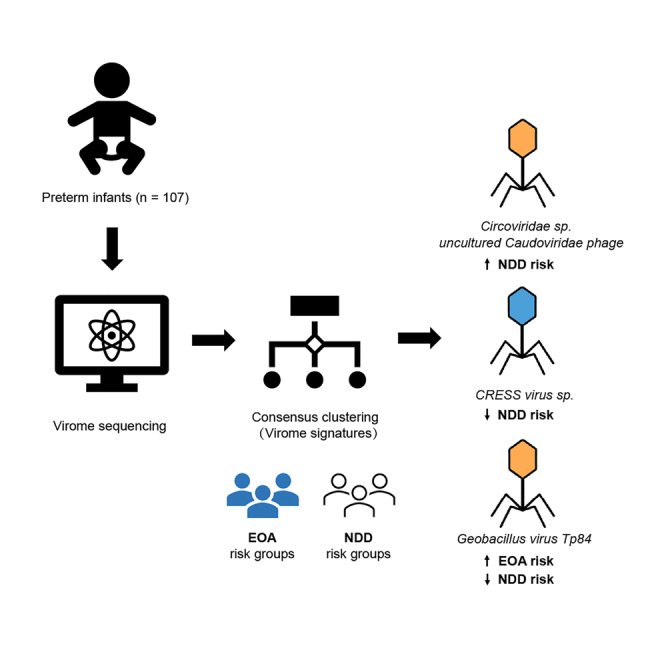

## Introduction

Preterm birth stands as a prominent global health concern, impacting a substantial portion of newborns globally, with an estimated 13.4 million newborns in 2020.^1^ These infants, born before the full maturation of their organs and systems, are at a heightened risk of various postnatal complications that can have profound implications for their health and well-being. These complications include necrotizing enterocolitis,^2^ early-onset anemia (EOA),^3^ and neurodevelopmental delay (NDD),^4^^,^^5^ collectively contributing to neonatal mortality and morbidity. The ramifications of these challenges extend beyond the short term, exerting lifelong impacts on the health, growth, and development of preterm infants.^6^^,^^7^

Notably, preterm infants born before 32 weeks of gestation exhibit significantly different patterns of intestinal microbial colonization compared to term infants.^8^ These atypical colonization patterns in preterm infants can elevate their vulnerability to infections and other systemic deficiencies.^9^ Bacteriophages are viruses that infect bacteria and play a role in regulating bacterial populations within a host ecosystem.^10^^,^^11^ Recent research has indicated that the infant’s gut virome is initially colonized by temperate bacteriophages induced by pioneer bacteria, followed by lytic bacteriophages and viruses that replicate in human cells.^12^ As previous studies have noted, preterm infants have compromised and underdeveloped gut barrier function relative to term infants.^13^^,^^14^^,^^15^ This impairment may facilitate microbial translocation and immune activation, potentially contributing to reduced bacteriophage strain-level richness in their feces.^16^ Notably, a study on weaned piglets demonstrated that bacteriophage supplementation could enhance intestinal barrier function and increase gut microbial richness, suggesting a potential link between gut barrier integrity and virome richness.^17^ Furthermore, the altered composition of the gut virome has also been linked to adverse health outcomes in children.^18^^,^^19^^,^^20^

While investigations into the gut virome remain in their nascent stages, a recent study has identified specific viral signatures preceding the onset of necrotizing enterocolitis in preterm infants, emphasizing the critical role of bacteriophages in preterm gut health.^21^ Nevertheless, the microbial mechanisms underlying other developmental complications in preterm infants remain insufficiently understood. Considering this, we hypothesize that differences in gut microbial colonization types and bacteriophage profiles could serve as pivotal risk factors for poor development outcomes in preterm infants. To address this hypothesis, we conducted a comprehensive analysis of the gut virome and bacteriome in a cohort of 107 preterm infants who were born without clinical complications or diseases but remain at risk for various developmental issues. This study seeks to unravel the intricate relationship between microbial colonization, particularly bacteriophages, and the development of preterm infants, shedding light on potential avenues for early intervention and improved care.

## Results

### Gut virome and bacteriome profiling of preterm infants

In this study, we analyzed stool samples collected from a cohort of 107 healthy preterm infants, each of whom exhibited no clinical complications or diseases at birth (Figure S1). These samples were collected within the first week of life, and the characteristics and clinical records of these eligible preterm infants are summarized in Table S1. The gestational ages of these infants ranged from 26.86 to 36.90 weeks, with a mean gestation age of 33.72 weeks. It is worth highlighting that both mean birth weight and average head circumference were below clinical health standards for most of these preterm infants. Furthermore, indicators of anemia, such as hemoglobin and RBC counts, revealed significant reductions in 64 of these preterm infants.

*Meta*-virome sequencing was performed on all 107 samples to analyze virus-like particles (VLPs). Collectively, we obtained an average of 44.57 ± 27.21 million clean reads per sample after removing host sequences. This substantial effort resulted in a virome dataset, totaling 6.67 ± 4.07 gigabytes per individual sample (Table S2). Following the rigorous process of virome assembly, we generated an average of 105,074 ± 102,906 contigs per sample, with an average N50 length of 935 base pairs (Table S2). Remarkably, among these viral contigs, approximately 73.35% ± 15.12% were attributed to bacteriophages, while the remaining contigs were categorically designated as animal viruses. This result underscores the exceptional quality of the virome sequencing effort (Table S2). Annotation based on this dataset unveiled the predominant viral families present in the first week of life of these preterm infants, with representatives including *Siphoviridae*, *Myoviridae*, *Podoviridae*, *Iridoviridae*, and *Circoviridae* (Figure S2). These viral family distributions mirrored those observed in healthy term infants in prior research,^12^ although *Iridoviridae* and *Circoviridae* families exhibited relatively higher richness in preterm infants. Furthermore, our analysis led to the identification of 306 distinct viral species across all samples (Table S3).

Complementing our exploration of the virome, we conducted an analysis of the bacteriome in these 107 preterm infants. Initially, 10 samples underwent whole-genome shotgun metagenomic sequencing, facilitating a thorough examination of the quantitative associations between the virome and bacteriome (Table S4). This analysis revealed a generally robust correlation between the virome and bacteriome in these samples (Figure S3), a finding consistent with previous studies.^22^ Subsequently, an additional set of 77 samples underwent 16S rRNA gene sequencing to provide a comprehensive view of the bacteriome composition of these infants (Tables S5 and S6). Collectively, this analysis led to the identification of a total of 13,025 amplicon sequence variants (ASVs) originating from the stool samples of preterm infants (Table S7).

### Classification of preterm populations based on virome signatures

**Figure 1:**
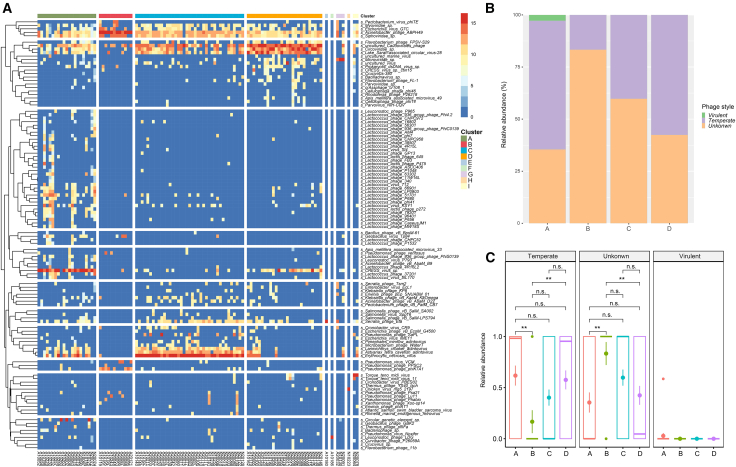
Characteristics of Gut Virome in Preterm Infants After Birth (A) Virome sequencing of fecal samples from 107 preterm infants within the first week of life revealed a total of 306 identified viral species. (B) Relative abundance of bacteriophages with different lifestyles at the species level among the groups. (C) Comparison of temperate, virulent, and unknown bacteriophages among the groups separately. The statistical significance for comparisons between groups was assessed by the Mann-Whitney U test (Group A: *n* = 21; Group B: *n* = 12; Group C: *n* = 39; Group D: *n* = 27). ∗∗*p*-value <0.01, n.s. indicates no significant.

Subsequently, we harnessed the power of consensus clustering analysis to effectively classify the preterm populations based on the relative abundances of viral species. The most robust clustering results emerged when the cumulative distribution function (CDF) reached a zenith at K = 9, characterized by a relatively gentle CDF descending slope and a compact area under the curve (AUC) (Figures S4A–S4C). With K = 9, the consensus matrix uncovered a total of four major groups, each consisting of more than 10 samples per group, alongside five smaller marginal groups, each comprising 1–3 samples per group. Hierarchical classification tree analysis (GraPhlAn) delved deeper, revealing that the viruses within these four major groups predominantly belonged to 7 phyla (*Nucleocytoviricota*, *Uroviricota*, *Cressdnaviricota*, *Phixviricota*, *Artverviricota*, *Cossaviricota*), 6 families (*Iridoviridae*, *Myoviridae*, *Siphoviridae*, *Podoviridae*, *Circoviridae*, *Adintoviridae*), 6 genera (*Otagovirus*, *Certrevirus*, *Skunavirus*, *Saundersvirus*, *Bruynoghevirus*, *Gemaleyavirus*) and 4 species (*Erythrocytic necrosis virus*, *uncultured Caudovirales phage*, *Acinetobacter phage ABPH49*, *Lake Sarah associated circular virus 28*) (Figure S5A). Furthermore, the distribution of viruses in these groups demonstrated distinct patterns, with Groups A, C and D being enriched in *Nucleocytoviricota*, *Uroviricota*, and *Cressdnaviricota*, while Group B exhibited exclusive enrichment in *Uroviricota*. The most abundant viral families within each group were *Siphoviridae* (Group A), *Myoviridae* (Group B), *Iridoviridae* (Group C), and *Circoviridae* (Group D), collectively accounting for more than 75% of all groups (Figure S5B). Nevertheless, the disparities in viral species composition were more pronounced than those at the family level across these groups, with key viral species identified as *CRESS virus* sp. (Group A), *Acinetobacter phage ABPH49* (Group B), *Erythrocytic necrosis virus* (Group C), and *uncultured Caudovirales phage* (Group D) (Figure 1A). The bacteriophage lifestyle prediction by detecting the integrases in viral contigs showed the abundance of temperate bacteriophage ranged from 16.67% to 61.76% among these four groups, and the virulent bacteriophage was only detected in Group A (Table S8). In the current analysis, 33 temperate bacteriophages were identified as temperate, while only 1 bacteriophage was recognized as virulent. The remaining 272 viral species are assigned to unknown, maybe due to an insufficient completeness of viral contigs. The analyses of relative abundance showed that temperate bacteriophages were more abundant in Group A and Group D rather than Group B (all *p*-values <0.01, Figures 1B and 1C).

### Composition and dynamics of gut virome are associated with early onset anemia

**Figure 2:**
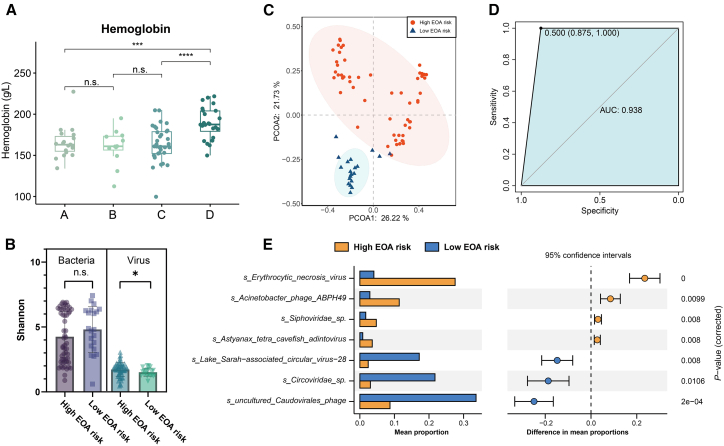
Association between Gut Virome and Early-Onset Anemia (EOA) in Preterm Infants (A) Comparative analysis of hemoglobin levels, with marked differences between the low EOA risk group (Group D) and the combined high EOA risk groups (Group A + B + C). (B) Alpha diversity analysis revealed a significantly higher Shannon index in the high EOA risk group compared to the low EOA risk group in the virome but not in the bacteriome. (C) Beta-diversity analysis unveiled a clear separation along Principal Component Analysis (PCoA) axis 2 in the virome between high and low EOA risk groups. (D) Validation of predictive capabilities for EOA risk using a random forest model trained on a dataset of more than 60 samples, with an Area Under Curve (AUC) value of 0.857, indicating promising validation for predicting EOA risk. (E) Differentially abundant viral species identified by STAMP analysis show 4 positively and 3 negatively associated viral species in the high EOA risk group relative to the low EOA risk group. The statistical significance for comparisons between groups in panels (A) and (D) was assessed by the Mann-Whitney U test. Differentially abundant viral species in panel (E) were identified using STAMP analysis (Group A: *n* = 21; Group B: *n* = 12; Group C: *n* = 39; Group D: *n* = 27; high EOA risk group: *n* = 72; low EOA risk group: *n* = 27). Statistical significance levels: ∗*p*-values <0.05, ∗∗∗*p*-values <0.001, ∗∗∗∗*p*-values <0.0001, n.s. indicates no significant.

We then thoroughly analyzed various hematological parameters in preterm infants among the four virome subtypes. Our findings unveiled noteworthy variations in hematological parameters, with substantial differences observed between Group D and the other three groups, while no statistically significant distinctions emerged among these three groups (Figure 2A; Figure S6A, *p*-value <0.001). Notably, the median value of minimal hemoglobin within the first week (137.5 g/L) in the three groups (Group A + B + C) aligned with a characteristic indicative of a high risk of early onset anemia (EOA) in preterm infants. In contrast, Group D (162 g/L) exhibited consistently elevated levels of hematological parameters, implying a potentially lower risk of developing EOA in this subgroup. Furthermore, the high EOA risk group (Group A + B + C, 54/72, 75%) showed a significantly higher incidence of EOA than the low EOA risk group (Group D, 7/27, 25.9%) (Figure S6A, *p*-value <0.001).

The analysis of alpha diversity in the virome showed a significantly higher Shannon index in the high EOA risk group compared to the low EOA risk group, indicating greater VLP diversity in the former (Figure 2B, *p*-value <0.05). However, no significant differences in alpha diversity were observed in bacterial communities between these two subtypes (Figure 2B; Figure S6B). Subsequent analysis of beta diversity also unveiled a pronounced segregation along Principal Coordinate Analysis (PCoA) axis 2 within the virome between the high and low EOA risk groups, while no substantial disparities were discerned in bacterial communities (Figure 2C; Figure S6C). Network analysis of viral co-occurrence in the low and high EOA risk groups exposed to a more tightly connected network with a reduced network size in the high EOA risk group (Figure S6D). These results strongly suggest the composition and dynamics of the gut virome may play a pivotal role in mitigating the risk of EOA in preterm infants.

Next, we delved into the potential association between specific VLPs and the occurrence of EOA in preterm infants. To identify the specific VLPs associated with EOA, we compared the virome of the low EOA risk group with the virome of the high EOA risk group and identified specific viral species that exhibited significant distinctions between the high and low EOA risk groups. The high EOA risk group featured 4 positively correlated VLPs and 4 negatively correlated VLPs relative to the low EOA risk group (Figure 2E; Figure S6E). These VLPs encompassed both animal viruses and bacteriophages, potentially serving as valuable biomarkers for predicting EOA risk in preterm infants.

To validate the predictive capabilities of these VLPs for EOA risk, we developed a random forest model trained on a dataset comprising over 60 samples, subsequently screening out 12 representative VLPs. Remarkably, all seven differential viral species were encompassed within these 12 representative VLPs (Figure S7). The validation process on the remaining samples within the testing set yielded an AUC value of 0.857, underscoring the significance of these VLPs as promising biomarkers for predicting EOA risk (Figure 2D; Figure S8). The Replication and repair pathway was significantly enriched in the low EOA risk group (Figure S11A). These findings emphasize the potential relevance of specific VLPs in the context of EOA and underscore the critical importance of considering gut virome in assessing EOA risk in preterm infants.

### Growth parameters of preterm infants are associated with gut virome composition

**Figure 3:**
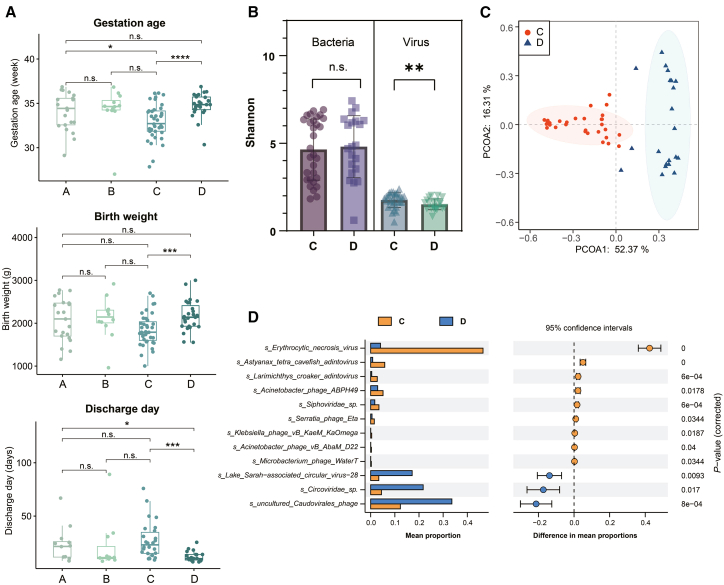
Association between Gut Virome and Growth Parameters of Preterm Infants (A) Comparative analysis of representative growth-related parameters, including gestational age, birth weight, and discharge days, reveals significant differences between Group C and Group D. (B) Alpha diversity analysis, indicating a significantly higher Shannon index in Group C compared to Group D in the virome, but not in the bacteriome. (C) Beta-diversity analysis showed a clear separation along Principal Component Analysis (PCoA) axis 1 in the virome between Group C and Group D. (D) Differentially abundant viral species identified by STAMP analysis show 10 positively and 3 negatively associated viral species in Group C relative to Group D. The statistical significance for comparisons between groups in panels (A) and (B) was assessed by the Mann-Whitney U test. Differentially abundant viral species in panel (D) were identified using STAMP analysis (Group A: *n* = 21; Group B: *n* = 12; Group C: *n* = 39; Group D: *n* = 27). Statistical significance levels: ∗*p*-value <0.05, ∗∗*p*-value <0.01, ∗∗∗*p*-value <0.001, ∗∗∗∗*p*-value <0.0001, n.s. indicates no significant.

We then analyzed the growth-related parameters in infants across the four groups, with notable differences emerging between Group C and Group D. Group C exhibited significantly lower values in growth-related parameters such as gestational age and birthweight, but significantly more discharge days when compared to Group D, making it an ideal comparator for identifying specific virome species correlated with the growth and development of preterm infants (Figure 3A, *p*-value <0.001).

Alpha diversity analysis cast further light on these distinctions, unveiling a significantly higher Shannon index in Group C relative to Group D (Figure 3B, *p*-value <0.01), although no significant differences were observed in the Simpson index (Figure S9A). These alpha diversity differences did not manifest in bacterial communities between these two subtypes (Figure 3B). Similarly, beta diversity analysis underscored a clear separation along PCoA axis 1 within the virome (Figure 3C), while no significant differences were discerned in bacterial communities (Figure S9B). Collectively, these results underscore the pivotal role of gut virome in shaping the growth and development trajectories of preterm infants.

Moreover, our investigation unearthed viral and bacterial species that significantly distinguished these two groups. Group C featured 10 positively correlated and 3 negatively correlated differential viral species in comparison to Group D (Figure 3D). Of these 13 viral species, four belonged to the realm of animal viruses, while the remaining nine species found their classification among bacteriophages. The Two-component system pathway was enriched in Group C, while Replication and repair were enriched in Group D (Figure S11B). In addition to viral species, our investigation also unveiled four bacterial species that significantly distinguish between these two groups (Figure S9C). These findings collectively present the possibility of using specific viral species as potential biomarkers associated with the growth and development of preterm infants.

### Pairwise comparison to find out key virus-like particles related to neurodevelopmental delay

**Figure 4:**
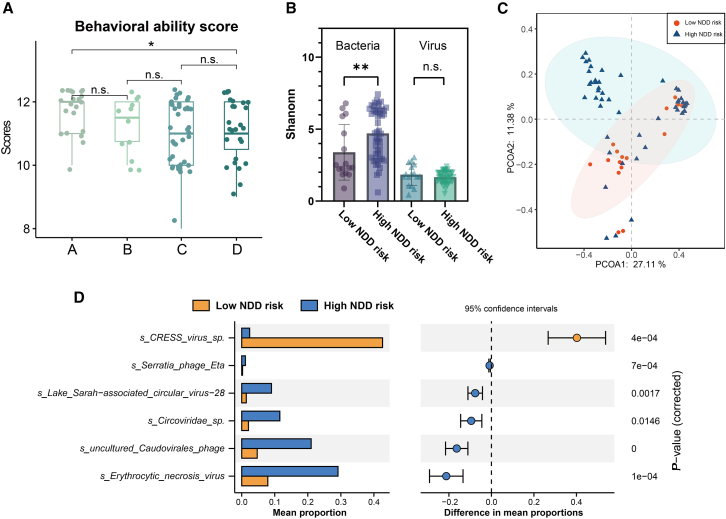
Association between Gut Virome and Neurodevelopmental Delay (NDD) in Preterm Infants (A) Comparative analysis of behavioral ability scores, highlighting marked differences between the low NDD risk group (Group A) and the combined high NDD risk groups (Group C + D). (B) Alpha diversity analysis, demonstrating a significantly higher Shannon index in the high NDD risk group compared to the low NDD risk group in the bacteriome but not in the virome. (C) Beta-diversity analysis revealing a distinct separation along Principal Component Analysis (PCoA) axis 2 in the virome between high and low NDD risk groups. (D) Differentially abundant viral species identified by STAMP analysis show 1 positively and 5 negatively associated viral species in the high NDD risk group relative to the low NDD risk group. The statistical significance for comparisons between groups in panels (A) and (B) was assessed by the Mann-Whitney U test. Differentially abundant viral species in panel (D) were identified using STAMP analysis (Group A: *n* = 21; Group B: *n* = 12; Group C: *n* = 39; Group D: *n* = 27; high NDD risk group: *n* = 66; low NDD risk group: *n* = 21). Statistical significance levels: ∗*p*-value <0.05, ∗∗*p*-value <0.01, n.s. indicates no significant.

To identify potential risk factors for NDD at postnatal 42 days, multiple linear regression analysis was performed. Gestational age was identified as a protective factor, while ABO blood type, mother’s age, antibiotic uses, platelet count, and Glucose-6-Phosphate Dehydrogenase (G6PD) deficiency were identified as risk factors (Table S9). We also analyzed parameters associated with neurodevelopment in infants among these four virome subtypes. In Group A (low NDD risk), the behavioral ability scores were significantly higher compared to Groups C and D (high NDD risk) (Figure 4A, *p*-value <0.05), indicating a potential association between neurobehavioral ability and virome subtype.

Furthermore, alpha diversity analysis of bacterial communities showed a significantly higher Shannon index in the low NDD risk group compared to the high NDD risk group (Figure 4B, *p*-value <0.01), while no significant divergence was observed in the Simpson index (Figure S10A). However, no significant alpha diversity differences were observed in the virome between these two subtypes. Beta diversity analysis revealed a clear divergence in PCoA axis 2 in the virome (Figure 4C), while no significant differences were found in bacterial communities (Figure S10B). These results underscore the importance of gut virome in the NDD of preterm infants.

Subsequently, we identified viral species that significantly differed between these two subtypes. The high NDD risk group had one positively correlated and five negatively correlated differential viral species relative to the low NDD risk group (Figure 4D). Out of these six viral species, two belonged to animal viruses (*Erythrocytic necrosis virus* and *Lake Sarah-associated circular virus-28*), while the remaining four species belonged to bacteriophages (*CRESS virus* sp., *Serratia phage eta*, *uncultured Caudovirales phage*, *Circoviridae* sp.), suggesting potential viral biomarkers associated with NDD in preterm infants. Instead, no significantly different bacterial species were revealed (data not shown). The Two-component system pathway was significantly enriched in the high NDD risk group (Figure S11C).

## Discussion

Preterm infants confront a myriad of challenges due to their underdeveloped organs and systems, making them susceptible to various postnatal complications. These complications, including intestinal disorders, EOA and NDD, are among the leading causes of neonatal morbidity and mortality worldwide.^23^^,^^24^ Understanding the intricate factors that contribute to these complications is crucial for improving the care and outcomes of preterm infants. Previous studies investigated the role of microbiota in early stages of preterm infants over the past two decades.^25^^,^^26^ Recently, the bacteriophages have been proven to regulate bacterial abundance, diversity, and metabolism to maintain gut homeostasis and even human health.^27^^,^^28^ Our study delved into the relatively uncharted territory of gut virome in preterm infants and its potential implications for health and development. Specifically, we hypothesized that differences in gut microbial colonization types and bacteriophage profiles may serve as risk factors for poor neurodevelopment in preterm infants with EOA. This hypothesis is grounded in existing research that has shed light on the multifaceted connections between the gut microbiome, EOA, and neurodevelopmental outcomes.

Extensive research has spotlighted the critical role of maternal microbiota in shaping the infant gut microbiome. Studies indicate that the maternal gut microbiome can influence the initial colonization of an infant’s gut, with microbial transmission occurring during birth and through breastfeeding.^29^ A study found that the maternal vaginal microbiome contains biomarkers associated with preterm birth susceptibility, underscoring the potential impact of the maternal reproductive microbiome on infant health.^30^ Additionally, research has shown that mobile genetic elements from the maternal microbiome can shape infant gut microbial assembly and metabolism, indicating a genetic link between maternal and infant microbiota.^31^ Detailed mapping of Bifidobacterium strain transmission from mother to infant via a dual - based and metagenomic approach provides evidence of mother - to - infant microbial transmission at the strain level.^32^ The maternal gut microbiome during pregnancy has also been shown to influence the developing immune system of the infant.^33^ Our study delved into the relatively uncharted territory of gut virome in preterm infants and its potential implications for health and development. Specifically, we hypothesized that differences in gut microbial colonization types and bacteriophage profiles may serve as risk factors for poor neurodevelopment in preterm infants with EOA. This hypothesis is grounded in existing research that has shed light on the multifaceted connections between the gut microbiome, EOA, and neurodevelopmental outcomes.

In this study, we investigated the potential association between gut virome, particularly bacteriophages, and the occurrence of EOA in preterm infants, along with its implications on neurodevelopment. Our VLPs extraction methods revealed the characteristics of gut virome within the first week of life, which was previously recognized as dark matter. The diversity and composition of gut virome showed large heterogeneity in recent research.^34^^,^^35^^,^^36^ The bacteriophage lifestyles demonstrated that temperate phages dominated in the preterm infant at the early stage, and even less virulent phages than in the previous study.^12^^,^^36^ Our study is the first time to show the pattern of bacteriophage lifestyle in preterm infants, and this pattern is reversed in the gut microenvironment of adults.^37^

From the comparisons of growth-related factors and EOA risk separately, the results indicated that the preterm infants with longer gestational ages and greater birthweights have higher levels of hemoglobin at birth, which means these preterm infants have higher EOA risks. EOA is a common complication in preterm infants, particularly those with very low birth weight, and has been linked to a range of adverse outcomes, including gastrointestinal disorders and neurodevelopmental impairments.^38^^,^^39^^,^^40^^,^^41^ These two comparisons, EOA risk and growth status, shared the similar patterns of gut microbial and viral composition. The representative viral species associated with EOA risks also appeared in the comparisons of growth-related factors. Particularly, preterm infants with very low birthweight showed a predominance of *Acinetobacter phage ABPH49* and *Siphoviridae* sp., and lower abundance of *Circoviridae* sp. and *uncultured Caudoviridae phage*, suggesting that this virome profile may be linked to increased EOA risk. *Acinetobacter phage ABPH49*, *Siphoviridae* sp. and *uncultured Caudoviridae phage* were the members of *Caudoviricetes* class. Interestingly, the *Siphoviridae* sp., a virulent bacteriophage with dsDNA, mainly infects Enterobacteria to destroy the bacterial cells directly, which can be a successful candidate for phage therapy.^42^ Of note, KEGG pathway analysis revealed that the Replication and repair pathway was significantly enriched in both the low EOA risk group and the high growth group. This pathway likely reflects a more stable and metabolically active microbial environment. Previous metagenomic studies have shown that enhanced microbial replication and DNA repair functions are characteristic of a robust and resilient gut microbiome, particularly during the early colonization period.^43^^,^^44^ Such microbial functions may contribute to maintaining intestinal homeostasis and epithelial integrity, potentially reducing the risk of EOA and supporting postnatal growth. Lastly, there were three categories that can be defined by crosstalk of viral richness within these two comparisons, including low birthweight with high EOA risk, regular birthweight with high EOA risk, and high birthweight with low EOA risk. These categories also proved that the consensus clustering based on the characteristics of gut virome was a more effective way to refine the typing of preterm infants, thereby achieving precise management and treatment.

The interplay between viral diversity and risks of EOA and NDD in preterm infants could be delineated into three categories: high EOA risk with regular neurodevelopment, high EOA risk with elevated NDD risk, and low EOA risk with high NDD risk. Notably, unlike growth-related factors and EOA risk, no significant differences in viral diversity were observed between high and low NDD risk groups. Our study identified *CRESS virus* sp. as the predominant bacteriophage in preterm infants with high EOA risk but low NDD risk, suggesting a potential neuroprotective role. Conversely, the presence of *Erythrocytic necrosis virus* and *Serratia phage eta* in infants with both high EOA and high NDD risks may indicate a harmful virome signature. *Serratia phage eta*, a temperate phage within the *Siphoviridae* family, may be less effective at modulating the bacterial compositions via the lysogenic life cycle at the first colonization stage, potentially contributing to microbial instability and increased bacterial diversity in the preterm infants with NDD. Additionally, KEGG pathway analysis revealed that the Two-component system pathway was significantly enriched in the high NDD risk group. This system, widely distributed among bacteria, governs environmental sensing and stress response regulation, and has been implicated in bacterial virulence and host-microbe interactions.^45^ The enrichment of this pathway may suggest a dysregulated or stress-prone microbial environment, which could adversely influence the gut–brain axis. Prior studies have proposed links between microbial signal transduction activity and early neurodevelopmental outcomes, especially in the context of immune activation and inflammatory responses.^46^

Among the viral species identified, *Geobacillus virus Tp84* was the only bacteriophage that exhibited both temperate and virulent lifestyles, which had not been previously regarded as having a life cycle.^47^ *Geobacillus virus Tp84* is a member of the *Caudovirales* order, *Siphoviridae* family, and infects several *thermophilic Geobacillus*. A previous analysis revealed *Geobacillus virus Tp84* played a role in against Gram-positive and Gram-negative bacteria with full activity at 30–75°C.^48^ Its dual lifestyle may allow more flexible control over bacterial populations, potentially buffering against dysbiosis in the context of EOA without exacerbating NDD risk. Interestingly, in preterm infants with low EOA risk but high NDD risk *Circoviridae* sp. and *uncultured Caudoviridae phage* were prevalent. A previous study reported that some viruses of the *Circoviridae* family could invade the brains of viremic patients.^49^ Once *Circoviruses* infiltrate the brain, they could trigger acute central nervous system infections or affecting neurological development by enhancing the effects of other diseases and facilitating the entry of other pathogens in to the nervous system.^50^ These findings raise the possibility that certain bacteriophage profiles, while beneficial in one physiological dimension, may pose risks to another, underscoring the complexity of gut virome–host interactions in preterm infants.

In the current study, the growth, EOA risk, and NDD risk were not directly correlated at the clinical level. However, we postulated that the gut microbiome, particularly bacterial and bacteriophage communities, may exert significant regulatory influence over these relationships. Specifically, enriched *Circoviridae* sp. and *uncultured Caudoviridae phage* alongside reduced *CRESS virus* sp., were observed in preterm infants with low EOA risks but increased NDD risk, suggesting a trade-off between hematologic stability and neurodevelopment. Our findings align with recent research highlighting the predominance of temperate bacteriophages in the preterm infant gut, emphasizing the role of early virome colonization patterns in shaping health trajectories.^12^^,^^36^ Our data suggest that while individual risk domains, such as EOA or impaired growth, may present with specific microbial and virome signatures, certain viral taxa and microbial pathways may bridge these phenotypes and jointly influence developmental outcomes.

In summary, our study reveals distinct gut virome signatures associated with EOA and NDD risks in preterm infants. These findings underscore the importance of bacteriophage composition in early life and suggest that virome-informed risk stratification could support more precise neonatal care. Longitudinal and functional studies are needed to validate these associations and explore phage-based interventions for improving long-term outcomes.

### Limitations of the study

However, several limitations should be acknowledged. While virome sequencing provides valuable insights, it is constrained by the limited scope of current viral reference databases, making it difficult to interpret unknown or unclassified sequences lacking functional or taxonomic annotations. Additionally, functional predictions based on 16S data and inferred KEGG pathways are indirect and require validation through metagenomic or transcriptomic approaches. Lastly, as this study represents a snapshot in time, longitudinal follow-up will be essential to assess the temporal dynamics of the gut virome and its long-term impact on EOA and neurodevelopmental outcomes.

## Resource availability

### Lead contact

Further information should be directed to Fei Gao (email: flys828@gmail.com).

### Materials availability

This study did not generate new unique reagents.

### Data and code availability

• Virome, Metagenome, and 16S sequencing data have been deposited at the National Center for Biotechnology Information as PRJNA1078999 and are publicly available as of the date of publication.
• This article does not report original code.
• Any additional information required to reanalyze the data reported in this article is available from the lead contact upon request.

## Acknowledgments

This work was supported by the Agricultural Science and Technology Innovation Program of Chinese Academy of Agricultural Sciences (CAAS) under Grant (1102432100071000008) and the Teaching Reform Research Project of South China Hospital, Medical School, Shenzhen University (YXBJG202340). We have not been paid to write this article by a pharmaceutical company or other agency. All authors read and approved of the final article.

We thank the E-gene company for 16S and virome sequencing. We also thank Cong Liu for helping to establish the extraction method of VLPs. We thank Ling Deng for his precious advice on virome analysis. We also thank Ziyuan Wu for the assistance with the storage and DNA extraction of fecal samples.

## Author contributions

Conceptualization: F.G., Y.Z., X.S., and S.R.; investigation, validation, and resources: S.R., C.H., and S.F.; data curation, methodology, and formal analysis: D.Z., S.R., Y.F., and Q.H.; funding acquisition: F.G. and X.S.; supervision: F.G. and Y.Z.; writing: S.R., T.L., D.Z., F.G., and X.S.

## Declaration of interests

The authors report there are no competing interests to declare.

## STAR★Methods

### Key resources table

**Table:** 

REAGENT or RESOURCE	SOURCE	IDENTIFIER
**Biological samples**		

Human fecal samples	This paper	N/A

**Reagent**		

QIAamp Viral RNA Mini Kit	Qiagen	Cat#52904
REPLI-g Mini Kit	Qiagen	Cat#150023
DNA Clean&Concentrator	ZYMO research	Cat#D4014
KAPA HIFI Hot Start DNA Polymerase kit	Roche	Cat#09420398001
QIAamp DNA stool extraction kit	Qiagen	Cat#51604
AMPure XP beads	Beckman Coulter	Cat#A63881
TruSeq® DNA PCR-Free Sample Preparation Kit	Illumina	Cat#FC-121-3003

**Deposited data**		

Sequencing data	This paper	PRJNA1078999

**Software and algorithms**		

MEGAHIT v1.0.6	Li et al.^51^	github.com/voutcn/megahit
Prodigal v2.6.3	Hyatt et al.^52^	github.com/hyattpd/Prodigal
CD-HIT v4.8.1	Fu et al.^53^	github.com/weizhongli/cdhit
STAMP v2.1.3	Parks et al.^54^	github.com/KatherLab/STAMP
ConsensusClusterPlus v1.36.0	Wilkerson et al.^55^	github.com/renzhonglu/ConsensusClusterPlus
Vegan v2.5-3	Oksanen et al.^56^	github.com/vegandevs/vegan
LinkET	Github	github.com/Hy4m/linkET
randomForest v4.7-1	Breiman,^57^	stat.berkeley.edu/∼breiman/RandomForests/
pROC v1.18.0	Robin et al.^58^	github.com/cran/pROC
blastp v2.12.0+	Camacho et al.^59^	blast.ncbi.nlm.nih.gov/Blast.cgi
checkV v1.0.1	Nayfach et al.^34^	bitbucket.org/berkeleylab/checkv
QIIME2 v2018-8	Bolyen et al.^60^	qiime.org/index-qiime1.html
FLASH v1.2.7	Magoč and Salzberg^61^	github.com/krishnaroskin/FLASH
KofamKOALA	Aramaki et al.^62^	github.com/takaram/kofam_scan
Trimmomatic v0.38	Bolger et al.^63^	www.usadellab.org/cms/?page=trimmomatic
ggClusterNet package v1.1.2	Wen et al.^64^	github.com/taowenmicro/ggClusterNet
Gephi v0.10.1	Liu et al.^65^	github.com/gephi/gephi
R v4.3.1	CRAN	www.r-project.org/

### Experimental model and study participant details

Preterm infants were enrolled from the Foshan Maternal Children’s Hospital cohort (Guangdong, China). A total of 392 infants were initially screened between 2018 and 2019, and 107 infants met inclusion criteria (admission to NICU within 24 h, fecal samples collected within the first week, and absence of congenital or metabolic disorders). Gestational ages ranged from 26.9–36.9 weeks, and both sexes were included. Neurodevelopmental outcomes were evaluated by the Neonatal Behavioral Neurological Assessment (NBNA). All guardians provided written informed consent, and the study was approved by the Ethics Committee of Sun Yat-sen University, in accordance with the Declaration of Helsinki. Analyses were stratified by sex when possible; no consistent sex-specific associations were detected, which is acknowledged as a limitation for generalizability.

#### Study population

The Foshan Maternal Children’s Hospital cohort study was an on-going hospital-based prospective cohort study designed to investigate the incidence of EOA and neurodevelopmental disorders in very low birth weight preterm infants and low birth weight preterm infants over the long term. The participants were randomly recruited from the neonatal intensive care unit (NICU) in Foshan Maternal Children’s Hospital, Guangdong, China. The Foshan Maternal Children’s Hospital cohort was established in 2015, and the participants involved in this study were recruited from 2018 to 2019. Ethical approval was obtained from the Ethics Committee of Sun Yat-sen University and conducted in accordance with the Declaration of Helsinki. Written informed consent was obtained from all participants’ legal guardians to involve in this cohort study and collect fecal samples from the neonates within the first week of life.

All participants were recruited at birth and followed up at 2-3 days, 12-14 and 26-28 days after correcting gestational age. The present study recruited 392 participants who: 1) birth or transferred to neonatal intensive care unit within 24 h after birth; 2) get the heel blood sample in the first 24 h; 3) collect the fecal samples within 7 days after birth; 4) were free of diseases affecting growth and development (e.g. congenital intestinal obstruction), metabolic diseases (e.g. hypothyroidism) and teratogenic disease (e.g. Robin syndrome) and further excluded participants who: 1). missing the data to calculate the neonatal behavioral neurological assessment score (n = 251); 2) death during hospitalization or lost to follow-up (n = 34); Ultimately, 107 participants were included in current study.

#### Data and sample collection

All participants had completed clinical records, a series of physical examinations and laboratory measurements. Briefly, the characteristics were collected including gestational age, birth weight, maternal age, sex, model of delivery, the history of mother`s gestation, medication history etc. The baseline Hb concentrations of the participants were collected from the clinical records for once or more in one week from birth to the first 28 days after birth. The Fecal samples were collected by cryotubes then stored at -80°C until DNA extraction and following analysis.

Hematology tests were performed with 40 μL heel blood immediately after birth or within the first 24 hours, and within the day during hospitalization if necessary. The hematology test was performed by automated hematology analyzer XN-10 (the Sysmex Corporation, Japan). Meanwhile, serum samples were collected for biochemical analyses by Automatic Biochemical Analyzer (Beckman, USA) at the same time.

Neurobehavioral impairment was performed by trained physicians, using Neonatal behavioral neurological assessment (NBNA) within 28 days after correcting gestational age (40 weeks).^66^ NBNA test was developed from Brazelton’s Newborn Behavior Assessment Scale by Bao Xiulan in 1988.^67^ It has stability and reliability in Chinese infants and has been a general test in screening early neurobehavioral impairments for decades. NBNA includes five scores: behavioral ability (6 items), passive muscle tone (4 items), active muscle tone (4 items), original reflex (3 items), and general assessment (3 items). For each item, it can be scored on three levels (0, 1, 2). The highest NBNA score is 40, and newborns with a score below 37 are usually considered abnormal.^68^

#### Ethics statement

Ethical approval was obtained from the Ethics Committee of Sun Yat-sen University and conducted in accordance with the Declaration of Helsinki. Written informed consent was obtained from all participants’ legal guardians to involve in this cohort study and collect fecal samples from the neonates within the first week of life.

### Method details

Fecal samples were collected within 7 days after birth and stored at –80°C. Virus-like particles (VLPs) were isolated by centrifugation, filtration, nuclease treatment, and DNA extraction, followed by multiple displacement amplification and Illumina NovaSeq sequencing. Clean reads were generated with Trimmomatic, host DNA removed with Bowtie2, and taxonomic classification performed with Kraken2/Bracken. Assembly (MEGAHIT), ORF prediction (Prodigal), clustering (CD-HIT), and functional annotation (KofamKOALA, KEGG) were used for virome characterization. Phage lifestyles were inferred based on integrase signatures and validated with checkV. Parallel bacterial profiling was conducted by 16S rRNA sequencing (QIIME2, SILVA database) and shotgun metagenomics.

For statistical analysis, diversity indices were calculated in R (vegan), consensus clustering performed with ConsensusClusterPlus, and differentially abundant taxa identified with STAMP. Associations with clinical data were tested by Mann–Whitney U, chi-square, Wilcoxon rank-sum, and multivariable regression. Predictive modeling was carried out using random forest with 10-fold cross-validation, and model accuracy was evaluated by ROC analysis (pROC). Sample inclusion/exclusion followed predefined criteria; missing data were imputed using the mice package. Randomization, replication, and stratified analyses were applied where appropriate, in line with NIH and ARRIVE guidelines.

#### VLPs isolation, nucleic acid extraction, amplification and purification

Fecal VLP DNA extraction for gut virome and whole DNA extraction for bacteriome were performed for the fecal samples. VLPs were isolated and purified from fecal samples as described previously.^12^ In brief, roughly 200 mg of feces were diluted and homogenized in 10 mL of SM buffer (100 mM NaCl, 8 mM MgSO_4_, 100 mM Tris-HCl pH 7.5), spun down for 30 min (5,000 g, Eppendorf, Germany) and filtered through 0.45 μm and 0.2 μm PES filters (Sartorius, Germany). The filtrate was concentrated by using a 50 kDa Centrifugal Filter Unit (Millipore, USA), resuspended in 10 mL SM buffer, and centrifuged three times to concentrate to approximately 500 μL. Pierce universal nuclease (Thermo Fisher, USA) was used to treat the concentration to degrade non-encapsulated nucleic acids for 10 min at room temperature. Total viral nucleic acid was extracted from the concentrate treated with nuclease by using QIAamp Viral RNA Mini Kit (Qiagen, Germany) without carrier RNA. After VLPs isolation, the control spiking experiments were performed with bacteriophage lambda, the results showed that more than 85% of plaque forming units could be recovered using current methods.

Due to extracted viral nucleic acids were extremely rare and could not be measured, thereby the multiple displacement amplification needed to be done by using REPLI-g Mini Kit (Qiagen, Germany) according to the recommendation of manufacturer, then amplified the samples for 10 h at 30°C,5 min at 65°C in PCR machine (Biometra, Germany) in order to avoid amplification preferences. Afterwards, the PCR products were purified with DNA Clean&Concentrator (ZYMO research, USA). The concentrations of purified products were measured by Qubit 3.0 Fluorometer (Life Technology, USA).

#### Library construction of VLPs and virome sequencing

The VLPs libraries were constructed by using BGI platform and method as described previously.^69^ Briefly, 20 μL transposase mixed with 5 μL annealed adapter then incubated at 25°C for 1 h before library construction. 1 ng genomic DNA was added to the mixer containing transposase and annealed adapter and incubated at 25°C for 10 min, then mixed with 5 μL 5X NT Buffer to stop the reaction for 5 min at room temperature. The reaction product was amplified at 94°C 1 min,10 cycles of 94°C 10 s,62°C 30 s and 72°C 30 s,then 72°C 5 min by using KAPA HIFI Hot Start DNA Polymerase kit. The amplified product was purified by XP bead. The library quality was assessed by Qubit® 3.0 Fluorometer (Life Technologies, USA), and the insert size was detected by 2100 Bioanalyzer system (Agilent Technologies, USA) after diluting library to 1 ng/μL. Finally, the library was sequenced on a Nova platform (Illumina, USA) with 150-bp paired-end reads.

#### Virome assembly, taxonomy annotation and diversity analysis

The methods of bioinformatics analysis process were described previously.^70^ Paired-end reads were precisely allocated to the respective samples based on the unique barcode and primer sequences. Trimmomatic (v0.38) was utilized to produce high-quality clean reads by removing adaptor sequences and low-quality sequences (SLIDINGWINDOW:5:15, HEADCROP:3, AVGQUAL:15, LEADING:5, TRAILING:5, MINLEN:80), with subsequent removal of PCR duplications.^63^ To eliminate host DNA, clean reads were aligned against the human reference genome (hg38) using Bowtie2 (v2.4.5) and reads that failed to align were retained for downstream analysis. Taxonomic information was annotated to clean reads by using Kraken2 (v2.1.2) & Bracken (v2.8) with reference to the Kraken2 Virus database. Subsequently, the clean reads underwent contig assembly employing MEGAHIT (v1.0.6).^51^ Prodigal (v2.6.3) was used to predict open reading frames (ORFs), followed by alignment of clean reads to these ORFs to calculate the mapped read count.^52^ Subsequently, ORFs were clustered to eliminate redundancy, resulting in the construction of a set of representative genes using CD-HIT software (v4.8.1).^53^ To mitigate bias stemming from ORF size variation, both the length of ORF sequences and sequencing depth were included in the data normalization process before conducting statistical comparisons. The normalized abundance matrix was computed for all samples by using previously described methods.^71^ ORFs were annotated with functional information using the Annotation software KofamKOALA of Kyoto Encyclopedia of Genes and Genomes (KEGG) based on the KEGG database with default parameters.^62^ Significantly different KEGG pathways were identified between viral subgroups using STAMP (v2.1.3) with adjusted *p*-values < 0.05.^54^

The correlation analysis between virus and clinical data was performed using R package corrplot (v0.92). The subtypes of gut virome in preterm infants is the classification by k-means consensus clustering with the R package ConsensusClusterPlus (v1.36.0).^55^ Viral relative abundance was visualized by R package pheatmap (v1.0.12). Alpha diversity analysis, indicative of species diversity complexity within each sample, was performed utilizing the R package vegan (v2.5-3).^56^ The alpha diversity across different viral subgroups was compared by using Wilcoxon rank-sum tests. Mantel tests were conducted to assess the correlations between the composition of virome or bacteriome, and community alpha diversity, clinical characteristics by the LinkET package (https://github.com/Hy4m/linkET). Beta diversity was computed by using the Bray–Curtis distance metrics with the R package vegan (v2.5-3) The dissimilarities in community structure, as determined by beta diversity, were visualized through Principal Coordinate Analysis (PCoA) using the R package ape (v5.7). Significantly different viral species were identified between viral subgroups using STAMP (v2.1.3) with adjusted *p*-values < 0.05.

The R package mice (v3.15.0) were employed to impute missing values in the clinical variables.^72^ Subsequently, a multivariable linear regression model was applied to determine clinical variables independently associated with NBNA scores. Variables with *p*-values < 0.05 in univariate analyses were selected as potential independent clinical variables associated with neurodevelopmental delay in preterm infants.

Using viral species with relative abundance over 0.01% as features, the random forest modeling was conducted using the R package randomForest (v4.7-1), and the importance scores of various viral characteristics were obtained and sorted.^57^ Viral species with greater importance scores (Mean Decrease Accuracy) were chosen based on the random forest result for the discovery cohort. Then a 10-fold cross-validation was performed on the discovery cohort using different numbers of selected viral species. The optimal number of selected viral species was determined based on the lowest error rate observed during cross-validation. Finally, a prediction model was constructed using the selected viral markers and the random forest algorithm. Receiver operator characteristic (ROC) analysis, based on the test cohort data, was performed to assess the accuracy of the viral biomarkers with the R package pROC (v1.18.0).^58^

#### The prediction of bacteriophage lifestyle

The database of integrase and large serine recombinase protein families was built from NR database (May, 2023) using blastdb_aliastool command, then used to predict whether phage contigs within viral gene families were temperate or virulent through blastp (v2.12.0+).^59^ checkV (v1.0.1) was employed to assess the quality of virome-assembled viral contigs.^34^ Families where more than 90% of complete phage contig did not harbor an integrase were deemed virulent, whereas for temperate families of both complete and incomplete phage contigs were required to carry an integrase. The final phage lifestyle relative abundance was calculated using the reads coverage depth of Contigs.

#### Bacterial library construction and 16S rRNA gene sequencing

Bacterial DNA extraction from the fecal samples was carried out using the QIAamp Fast DNA Stool Mini Kit (Qiagen, Germany) following the manufacturer's instructions. The processes of bacterial library construction, 16S rRNA gene sequencing and data processing were described previously.^73^ The genomic DNA was subsequently analyzed by 1% agarose gel electrophoresis. Following this, all DNA samples underwent quantification by using a Qubit 3.0 Fluorometer (ThermoFisher Scientific, USA). Following that, PCR amplification targeted the V3-V4 hypervariable region of the 16S rRNA gene, employing the 338F forward primer (5’- ACTCCTACGGGAGGCAGCA-3’) and the 806R reverse primer (5’-GGACTACHVGGGTWTCTAAT-3’). The PCR cycling protocol consisted of initial denaturation at 94°C for 3 min, followed by 25 cycles of 94°C 45 s, 50°C 60 s and 72°C 90 s, with a 10 min final extension step at 72°C. Subsequently, the amplicon products underwent purification using AMPure XP beads (Beckman Coulter, USA). Sequencing libraries were then prepared using the TruSeq® DNA PCR-Free Sample Preparation Kit (Illumina, USA) following the manufacturer's instructions. The qualification of library was evaluated using the Agilent Bioanalyzer 2100 system (Agilent Technologies, USA). Following quality assessment, the high-quality libraries were sequenced on an Illumina NovaSeq 6000 platform, generating paired end reads with a length of 150 bp.

The raw pair-end reads were merged and overlapped using FLASH software (v1.2.7) to obtain raw tags.^61^ Trimmomatic (v0.38) was employed to filter out low-quality raw tags, retaining only high-quality clean tags. Subsequently, these clean tags were imported into the Quantitative Insights Into Microbial Ecology 2 (QIIME2 v2018-8) software package for further analysis.^60^ Within the QIIME2 environment, sequences underwent quality filtering and denoising using the Divisive Amplicon Denoising Algorithm 2 (DADA2) pipeline. The Taxonomy assignment was conducted using the 99% identity SILVA (release 132) V3-V4 classifier. All the 3,338 ribosomal sequence variants (RSVs) were identified as unique features across all samples without any clustering. The feature rooted phylogenetic tree, representative sequences, and metadata from QIIME2 were exported for further analysis in R software (v4.3.1). Additionally, alpha and beta diversity were computed using a rarefaction depth of sequences, which were a similar method with viral analysis described above. Significantly different bacterial species between subgroups were identified using STAMP (v2.1.3), with statistical significance set at adjusted *p*-value < 0.05.

#### Shotgun metagenome sequencing and data processing

Genomic DNA extraction from the fecal samples was carried out using the QIAamp Fast DNA Stool Mini Kit (Qiagen, Germany) following the manufacturer's instructions. Sequencing libraries were then generated using the TruSeq® DNA PCR-Free Sample Preparation Kit (Illumina, USA), which included the incorporation of index codes. The quality of the libraries was assessed using both the Qubit 3.0 Fluorometer (Thermo Scientific, USA), and the Agilent Bioanalyzer 2100 system (Agilent Technologies, USA). Following that, the high-quality libraries were sequenced on an Illumina NovaSeq 6000 platform, generating paired end reads with a length of 150 bp.

Trimmomatic (v0.38) was utilized to produce high-quality clean reads by removing adaptor sequences and low-quality sequences with the following parameters (TruSeq3-PE.fa:2:30:10:8:true AVGQUAL:15 LEADING:5 TRAILING:5 MINLEN:80). The Human Sequence Removal protocol provided by NCBI was utilized to remove human genome reads (hg38) from clean reads. The high-quality clean reads were subjected to two parallel downstream analyses: (1) For taxonomic profiling, the reads were annotated using Kraken2 (v2.1.2) and Bracken (v2.8) to determine the community composition; and (2) separately, the same set of clean reads was used for *de novo* genome assembly with MEGAHIT (v1.0.6) using the specific parameters '--k-min 21 --k-max 149 --k-step 10'. Prodigal (v2.6.3) was used to predict ORFs, and the clean reads were aligned to ORFs to calculate the mapped read count. Following that, the ORFs were clustered, and redundant sequences were eliminated to generate a set of representative genes by CD-HIT (v4.8.1). The normalized abundance matrix was computed using previously established methodologies.^70^

The gut microbiota data were subjected to statistical analysis by using the R package vegan (v2.5-3), which facilitated PCoA, alpha and beta diversity analysis, and Procrustes analysis. Co-occurrence networks were constructed using the SparCC method in R (v4.3.1) with the ggClusterNet package (v1.1.2).^64^ Significant correlations (magnitude > |0.3|, *p*-value < 0.05) were visualized in Gephi (v0.10.1).^65^

### Quantification and statistical analysis

Statistical analysis was performed using the Statistical Package for the Social Science (SPSS) (v22, IBM, Chicago, IL, USA). Quantitative variables were presented with mean and standard deviation. Categorical variables were presented with numbers and percentages. The Mann-Whitney U test was performed for the comparison of quantitative variables, while chi-square test was used for categorical variables, for the comparison between the high EOA risk group and low EOA risk group. Multiple linear regression was used for assessing the correlations between EOA and NDD in preterm infants, controlling for confounding factors (gestational age, birth weight, maternal age, antibiotics, and anemic status at discharge). The NBNA scores were treated as continuous variables. We initially ran a univariate analysis in model 1 and then adjusted for gestational age in model 2, birth weight in model 3. We further use a multiple linear regression for the stratified analysis by sex. Model 1, model 2, and model 3 are consistent but we did not perform model 4 due to the limited participants in the subgroup. *p*-value < 0.05 was considered statistically significant.

## Supplemental information

Document S1. Figures S1–S11

Table S1. Clinical characteristics in this study

Table S2. Virome sequencing data summary in this study

Table S3. Relative abundance of viral species in this study

Table S4. Relative abundance of bacterial species by metagenomic sequencing in this study

Table S5. 16S sequencing summary in this study

Table S6. Bacterial composition of 16S sequencing in this study

Table S7. ASV read count of metagenomic sequencing in this study

Table S8. Lifestyle of bacteriophages in this study

Table S9. Multiple linear regression analysis of clinical parameters in this study
