# Supplementary material for: Gut virome characteristics associated with early onset of anemia and neurodevelopmental delay in preterm infants
Source: iScience. 2025 Sep 16;28(10):113578. doi: 10.1016/j.isci.2025.113578 (PMC12510052; doi:10.1016/j.isci.2025.113578)
Supplement: Document S1. Figures S1–S11 [file mmc1.pdf]

iScience, Volume *28*

Supplemental information

Gut virome characteristics associated with early onset of anemia and neurodevelopmental delay in preterm infants

Shuqiang Ren, Du Zhang, Xingwei Shi, Tianze Li, Qi Hu, Yance Feng, Chenghao Hu, Siting Feng, Yanna Zhu, and Fei Gao

Figure S1

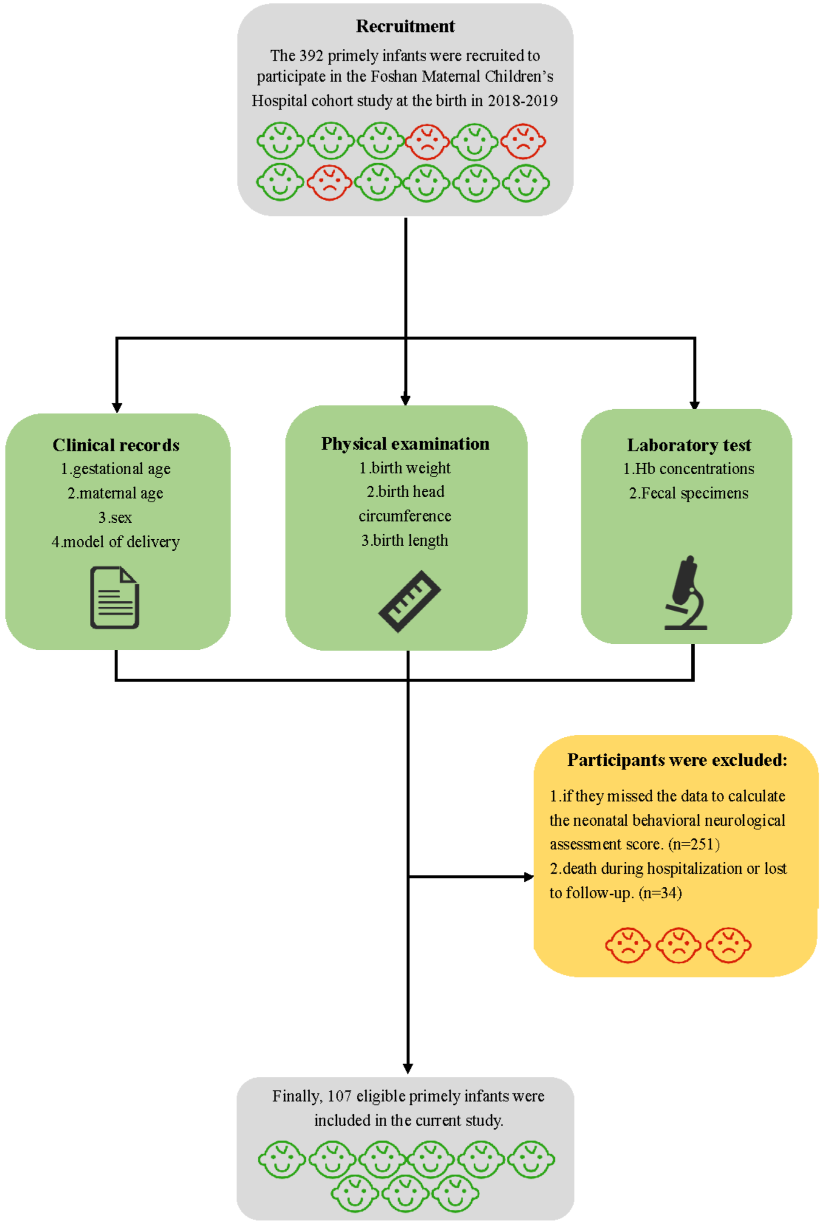

Supplementary Figure 1. The flow scheme of the study population in current analysis. 392 preterm infants were recruited in the current cohort from Foshan Maternal Children's Hospital in 2018-2019. All infants performed with clinical records, physical examination and laboratory test in hospital. Participants were excluded according to lack of NBNA score (n=251) and follow-up (n=34). Finally, 107 eligible preterm infants were included in the current analysis.

Figure S2

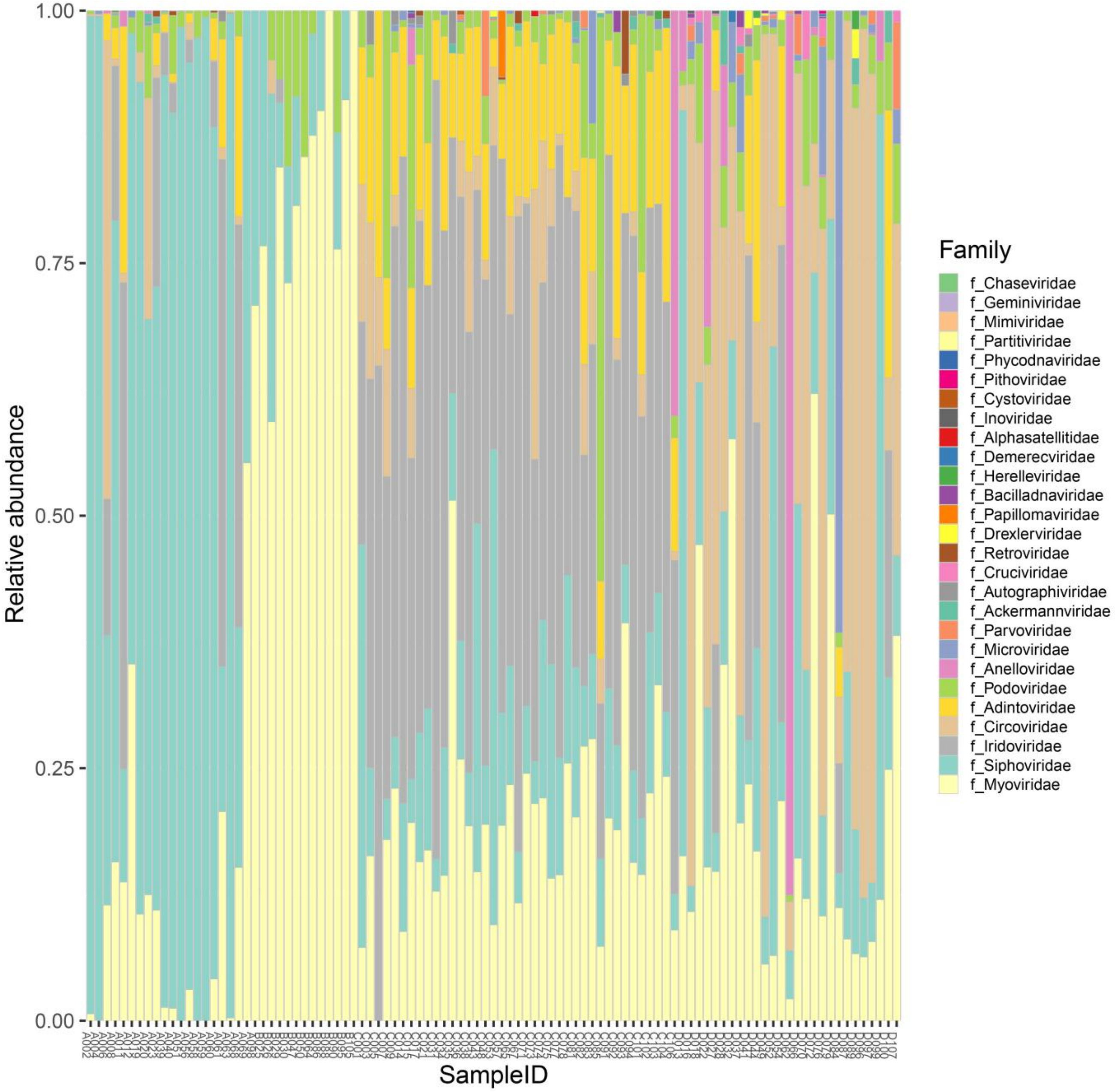

Supplementary Figure 2. The relative abundance of bacteriophages at family level in preterm infants. In the context of preterm neonates, the comparative prevalence of bacteriophages at the familial taxonomic level was examined. A total of 27 viral families, each exhibiting a relative abundance exceeding 1%, were discerned across a cohort comprising 107 samples from preterm infants.

Figure S3

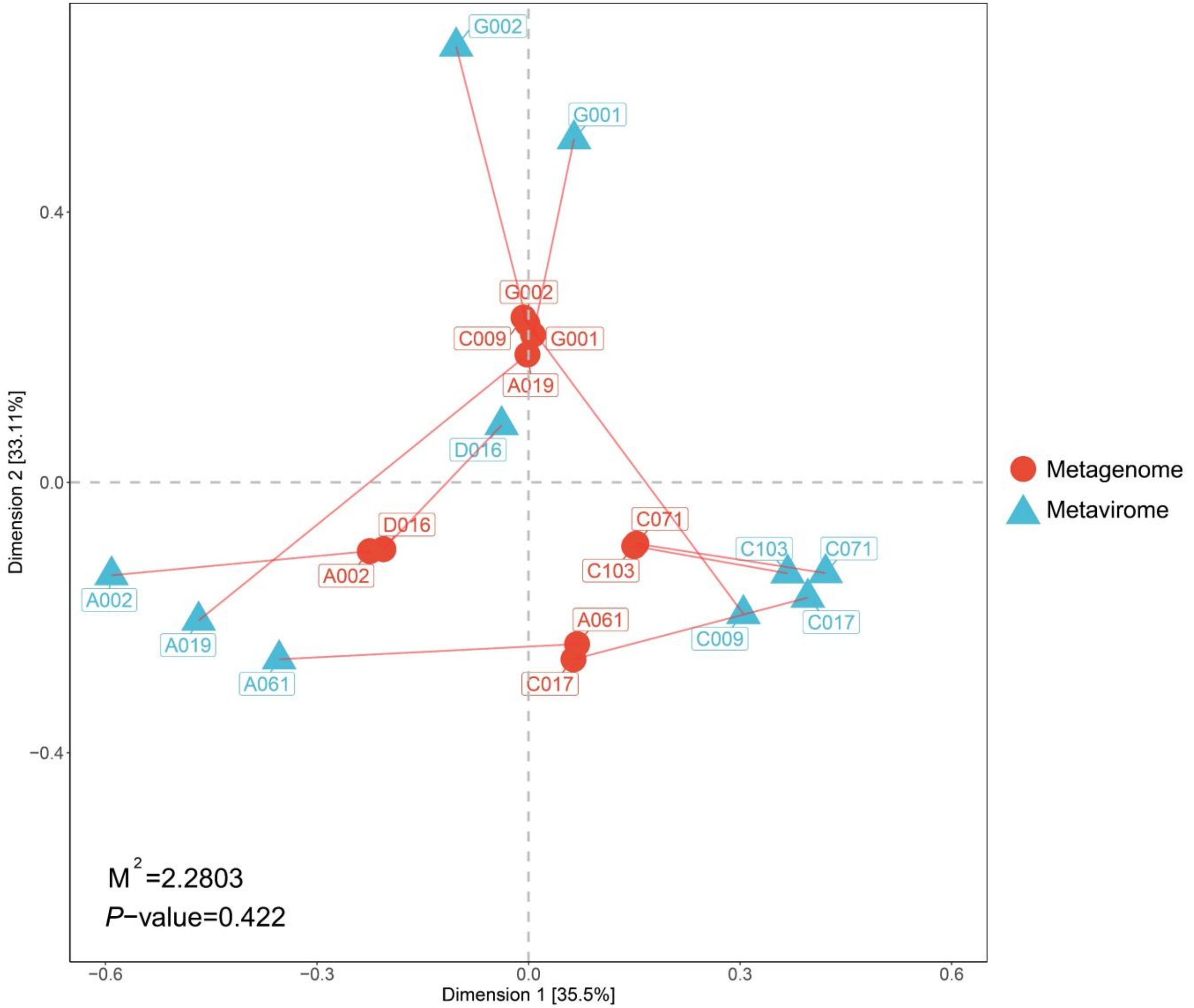

Supplementary Figure 3. The correlation between gut bacterial community and viral community in preterm infants. A comparative metagenome and virome analysis were conducted on a set of 10 fecal specimens. The co-occurrence patterns within these specimens were elucidated through the construction of a Pearson correlation matrix, a statistical method employed to assess the linear relationship between bacterial community (red circle) and viral community (blue triangle). This matrix was computed using the R software environment, which is widely recognized for its robust capabilities in statistical computing and graphics. Subsequently, the topological representation of these correlation networks was visualized using Gephi, a software tool designed for network modeling and analysis, thereby facilitating the exploration of complex network structures and their inherent dynamics.

Figure S4

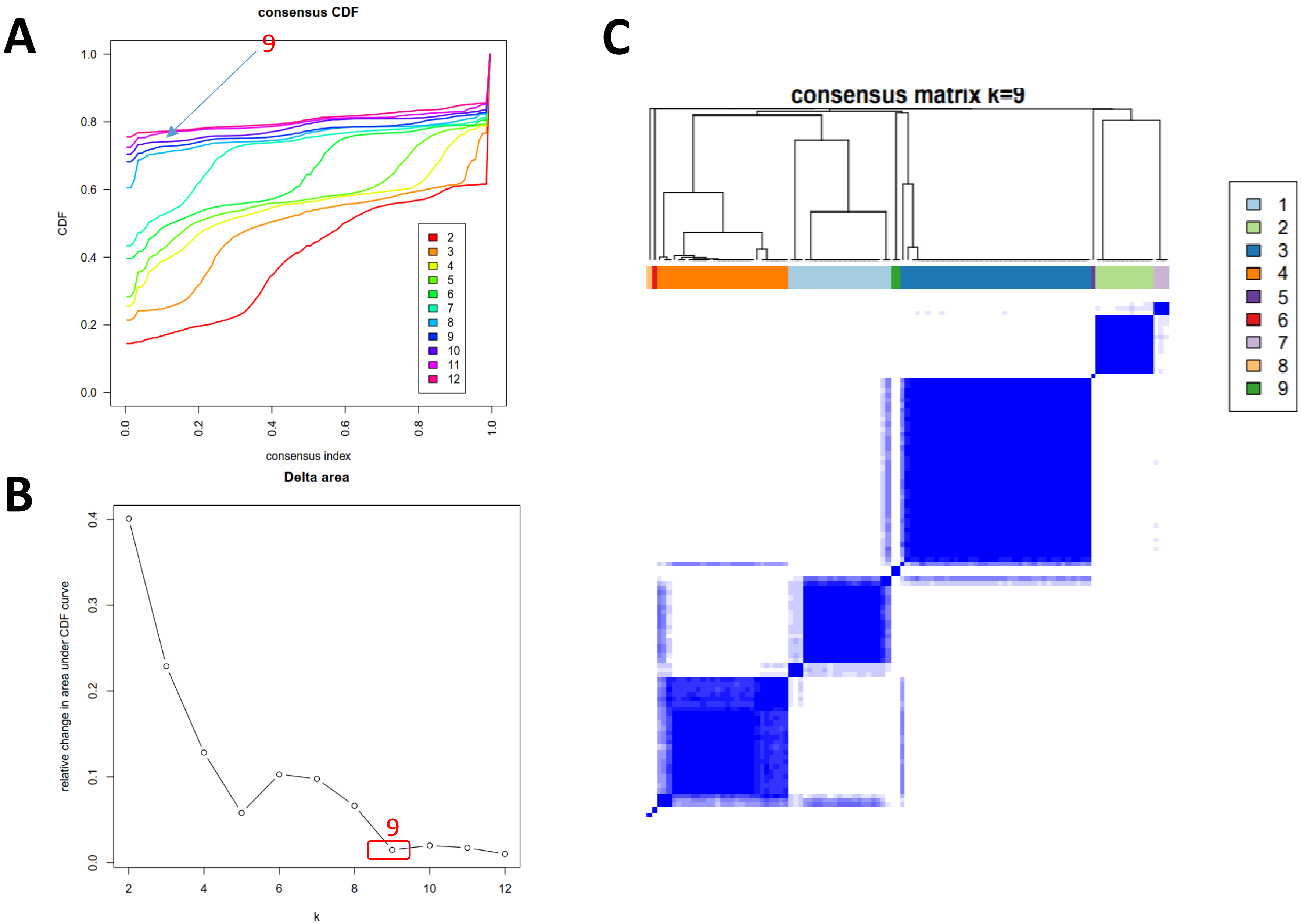

Supplementary Figure 4. Consensus clustering analysis based on the relative abundance of viral species. (A) When K=9, a range from 2 to 12, the slope stabilized and approached to 0, implying that K=9 is a reasonable grouping. (B) The relative change in area and CDF curve showed that K=9 had the minimized area under the curve. (C) A consensus clustering method showed the grouping of all samples when K=9.

Figure S5

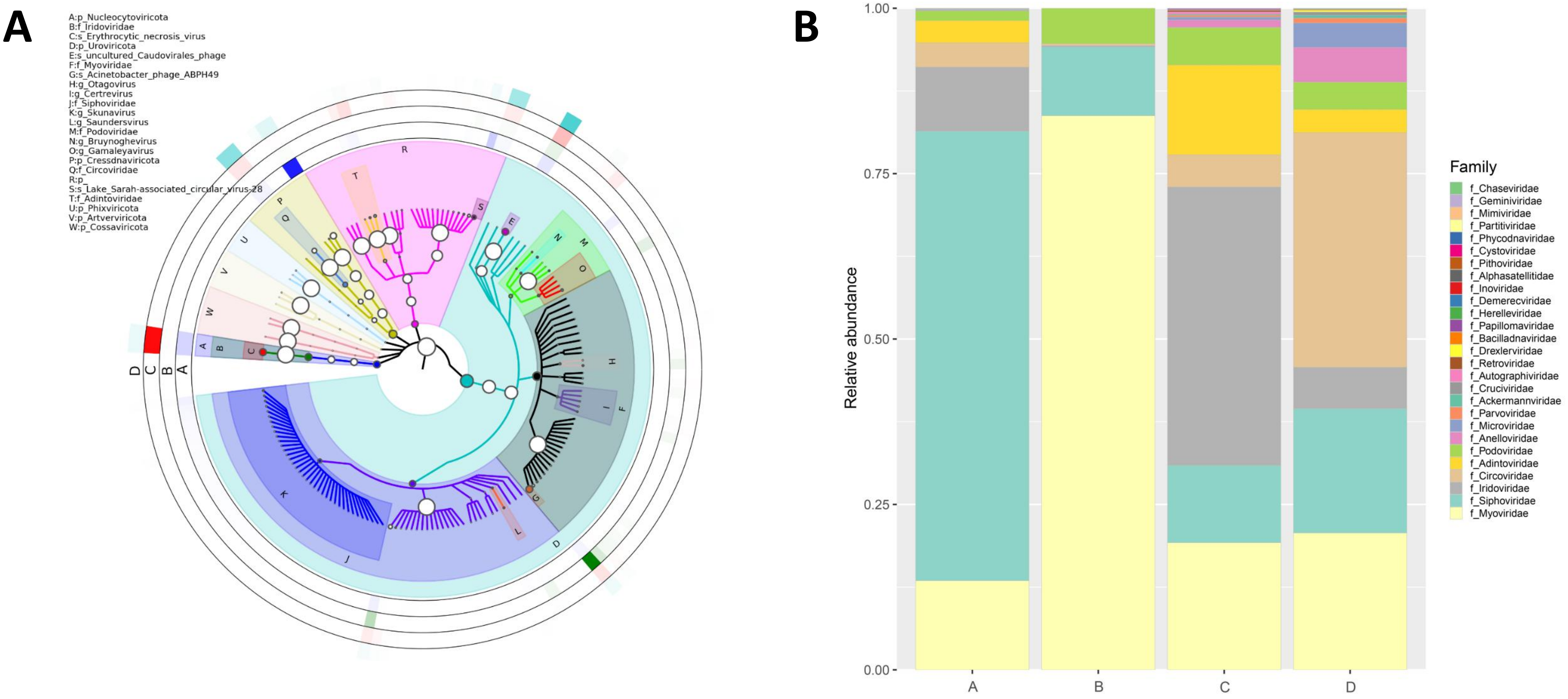

Supplementary Figure 5. Hierarchical classification tree analysis and the relative abundance of prevalent bacteriophages at family level within the four major groups. (A) This analysis involves the use of a tree-like model to classify data hierarchically. In the context of bacteriophages, this could involve classifying them based on genetic sequences, host specificity, or other phylogenetic markers. Progressing from the core (Group A) to the periphery (Group D), moving from the central aspects outward. (B) The relative abundance refers to the proportion of each bacteriophage family in the total community within the four major groups.

Figure S6

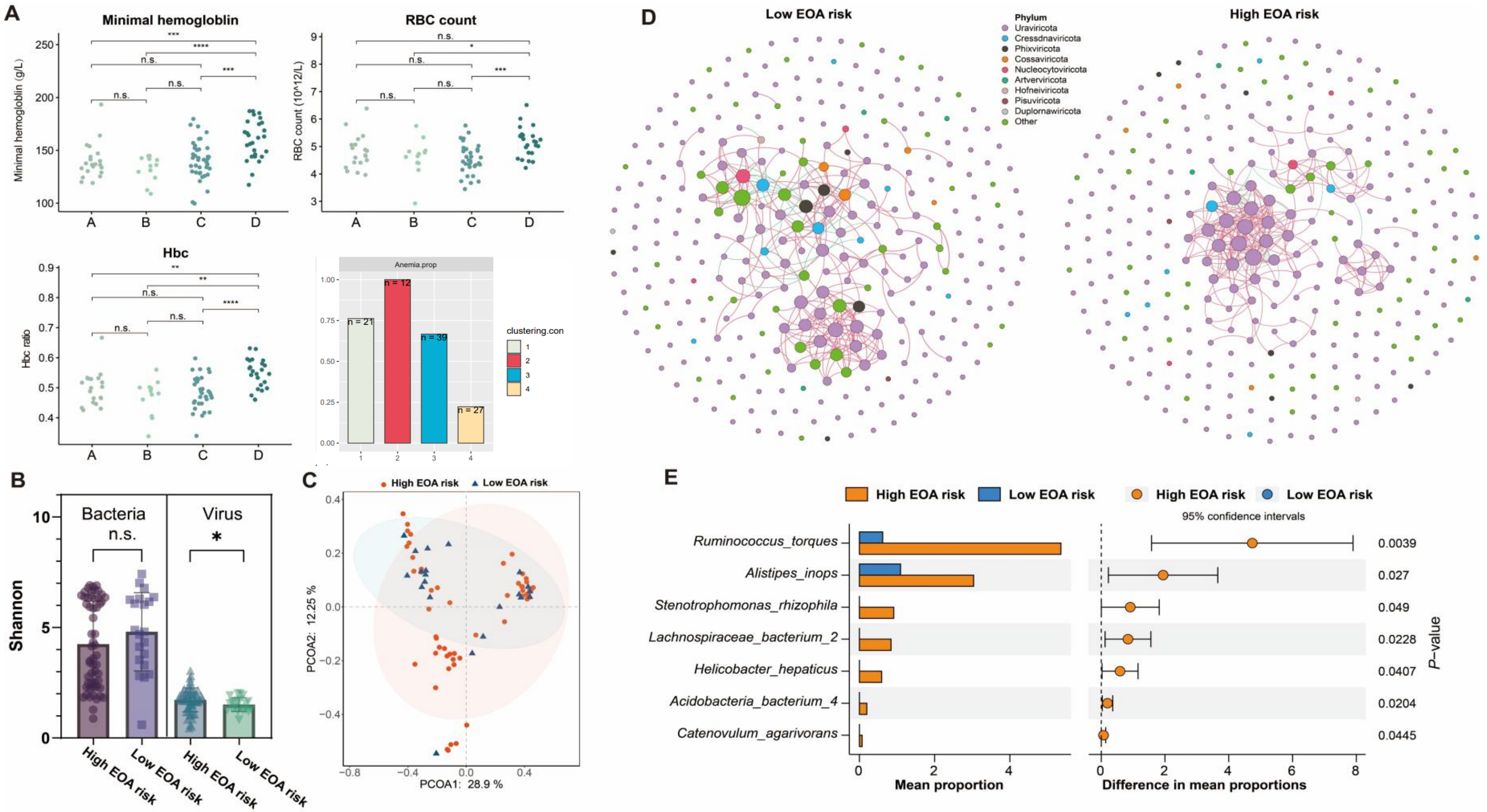

Supplementary Figure 6. The analysis of hematological parameters related to early onset anemia (EOA), bacterial community, and differential bacterial species. The analysis of hematological parameters related to early onset anemia (EOA), bacterial community, and differential bacterial species. (A) Comparisons of hematological parameters including minimal levels of hemoglobin, red blood cell count, hematocrit. (B) The analysis of alpha diversity showed a significant higher Shannon index between high (Group A+B+C) and low EOA risk group (Group D). (C) Beta-diversity analysis showed no differences between high and low EOA risk groups in the bacterial community. (D) Network analysis of viral co-occurrence in the low and high EOA risk groups, indicating a more tightly connected network with a reduced network size in the high EOA risk group. (E) Differentially abundant bacterial species identified by STAMP analysis show 7 positively associated bacterial species in the high EOA risk group compared to the low EOA risk group. The statistical significance for comparisons between groups in panels (A) and (B) was assessed by the Mann-Whitney U test. Differentially abundant viral species in panel (E) were identified using STAMP analysis (Group A: n = 21; Group B: n = 12; Group C: n = 39; Group D: n = 27; high EOA risk group: n= 72; low EOA risk group: n = 27). *p-value < 0.05, ***p-value < 0.001, ****p-value < 0.0001, n.s. indicates no significant.

Figure S7

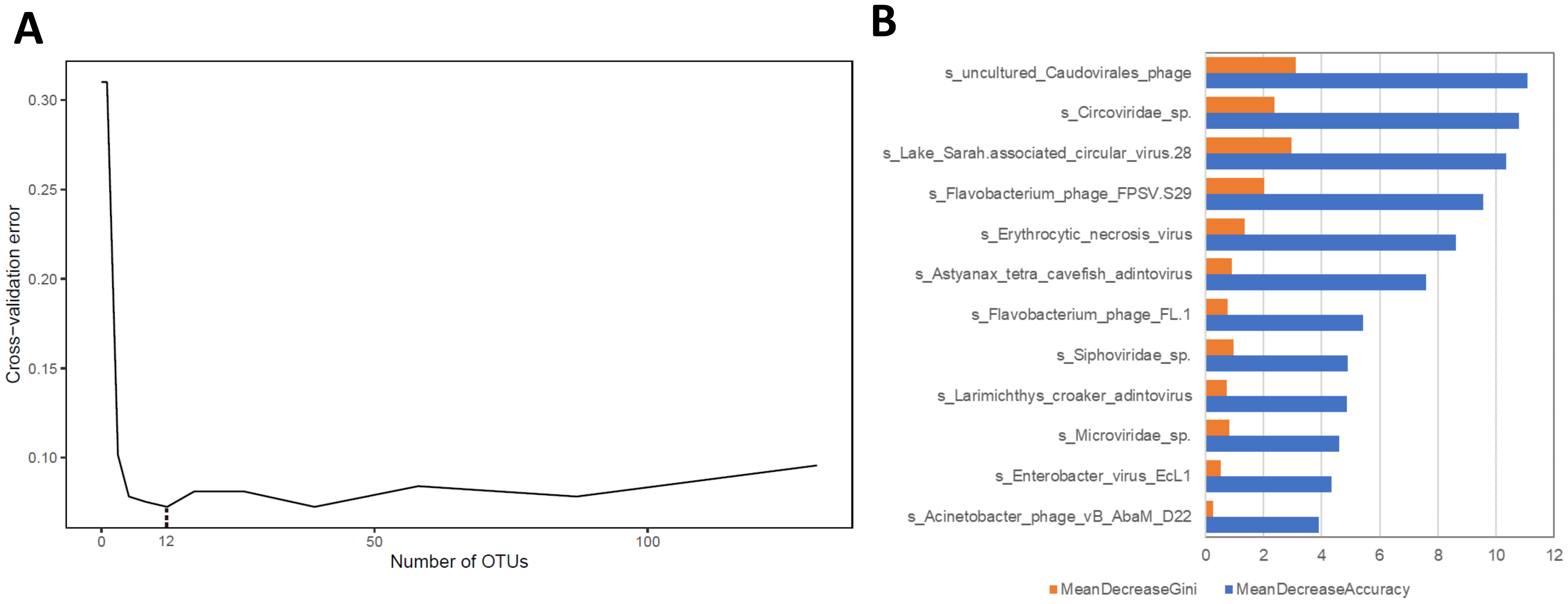

Supplementary Figure 7. The validation of the predictive capabilities of the VLPs for EOA risk by a random forest model, subsequently screening out 12 representative VLPs. (A) Viral species with greater importance scores (Mean Decrease Accuracy) were chosen based on the random forest result for the discovery cohort. (B) The optimal number of selected viral species (n=12) was determined based on the lowest error rate observed during cross-validation.

Figure S8

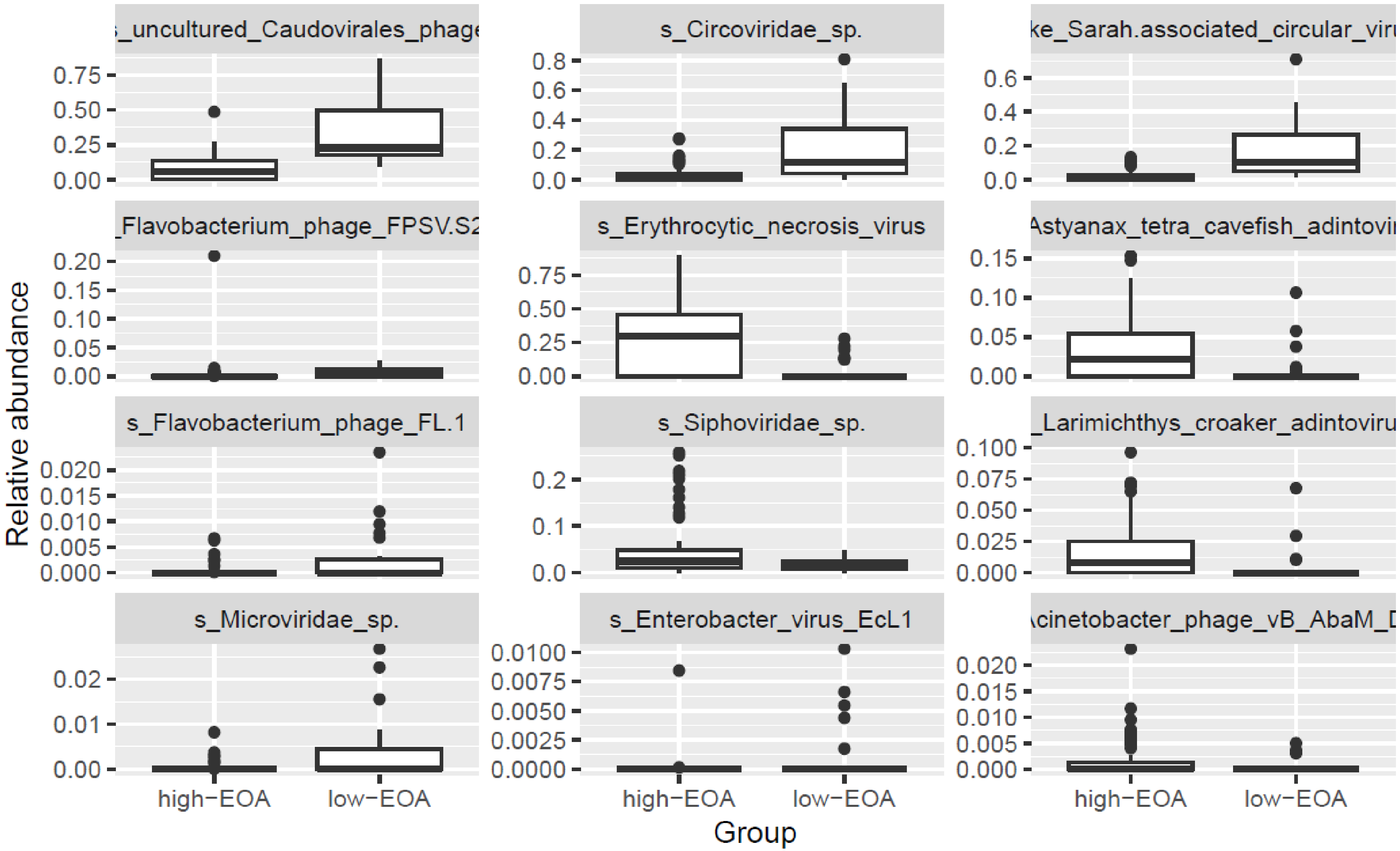

Supplementary Figure 8. The relative abundance of the representative 12 viral species between high and low EOA risk groups. Random forest trained data to compare the representative 12 viral species between high and low EOA risk groups.

Figure S9

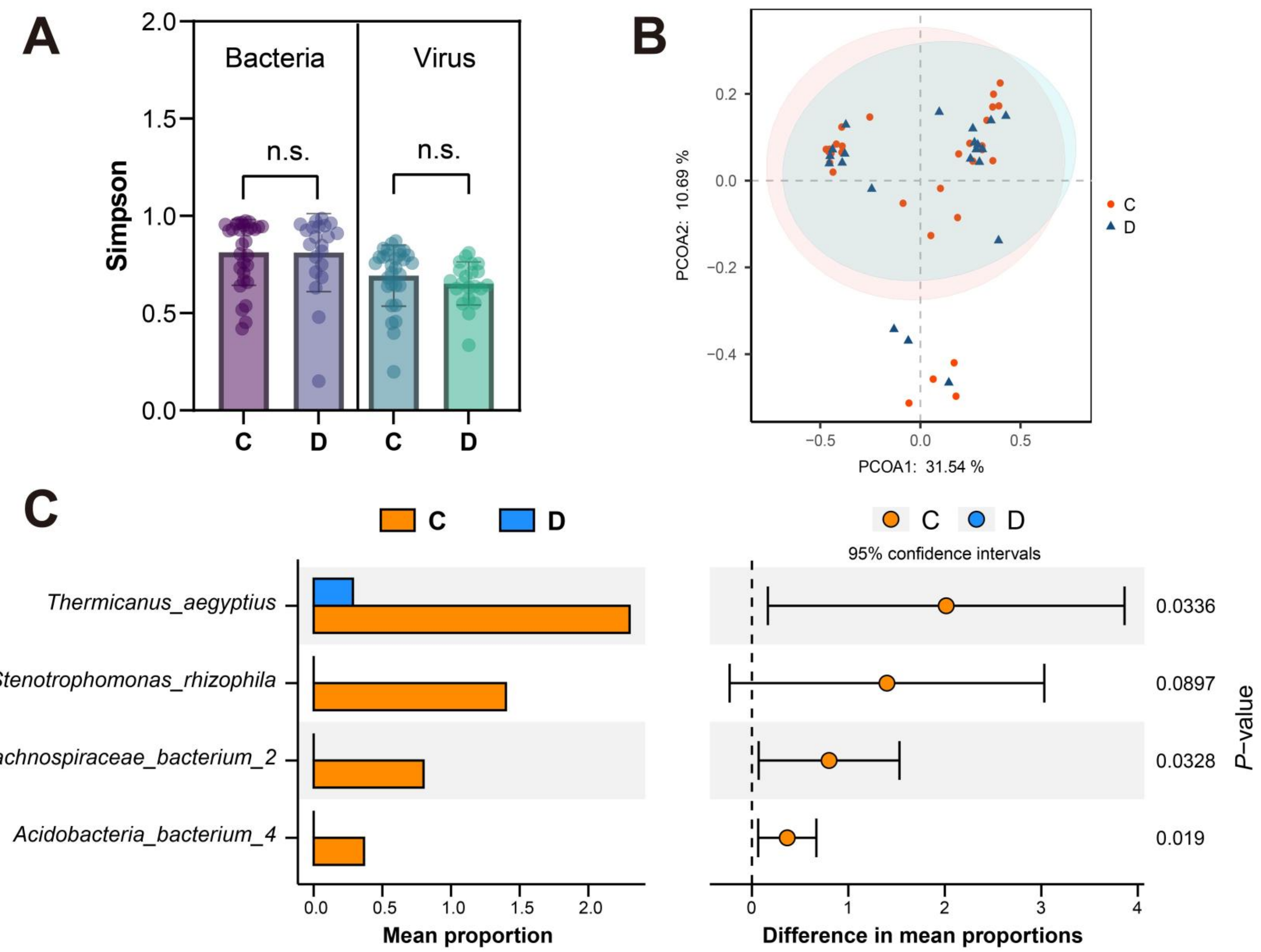

Supplementary Figure 9. The comparison of virome and bacteriome between Group C and Group D. (A) Alpha diversity analysis, indicating no significant Simpson index of both virome and bacteriome between Group C and Group D. (B) Beta-diversity analysis showed no separation along PCoA1 and PCoA22 in the bacteriome between Group C and Group D. (C) Differentially abundant bacterial species identified by STAMP analysis show 4 positively associated bacterial species in Group C relative to Group D. The statistical significance for comparisons between groups was assessed by the Mann-Whitney U test. (Group C: n = 39; Group D: n = 27). n.s. indicates no significant.

Figure S10

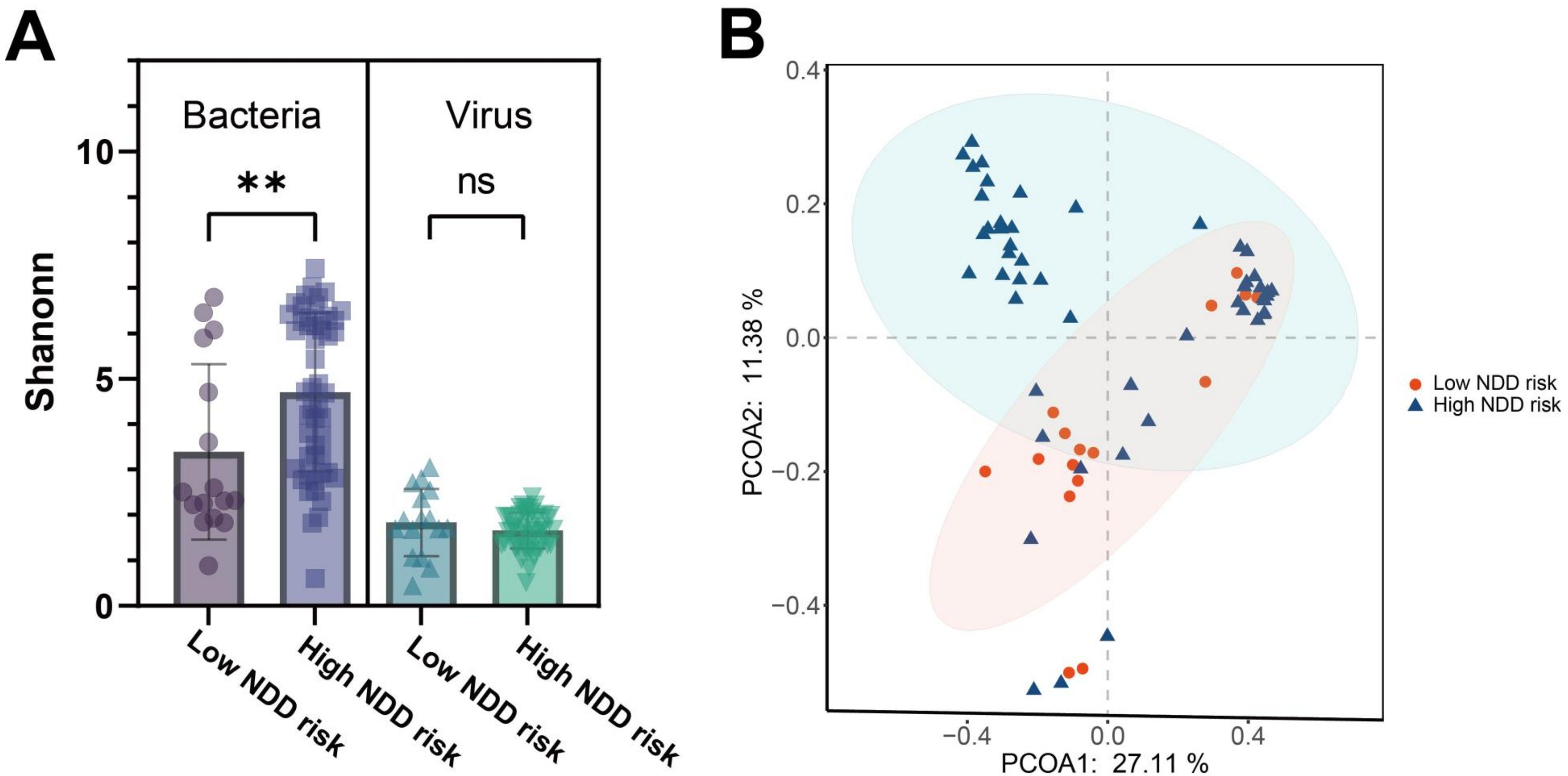

Supplementary Figure 10. The analysis of alpha and beta diversities between high and low NDD risk groups. (A) Alpha diversity analysis, indicating a significant Simpson index of bacteriome between high- and low-NDD risk groups. (B) Beta-diversity analysis showed no separation along PCoA1 and PCoA22 in the bacteriome between Group C and Group D. The statistical significance for comparisons between groups was assessed by the Mann-Whitney U test (high NDD risk group: n= 66; low NDD risk group: n = 21). **p-value < 0.01, n.s. indicates no significant.

Figure S11

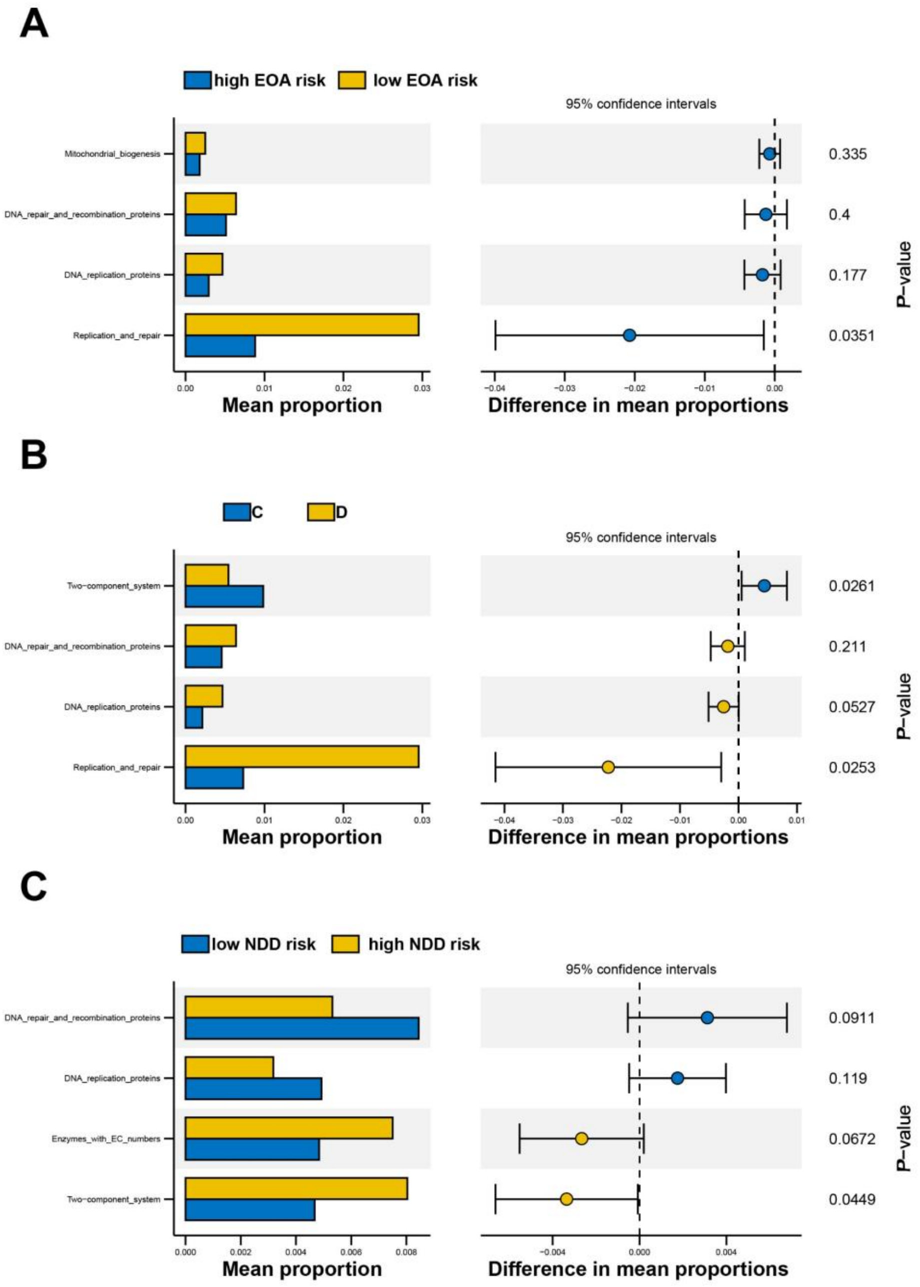

Supplementary Figure 11. Differentially enriched KEGG pathways identified by STAMP analysis across groups. Bar plots show significantly enriched functional pathways between (A) high vs. low EOA risk, (B) Group C vs. Group D, and (C) high vs. low NDD risk groups. Differentially abundant viral species were identified using STAMP analysis (Group C: n = 39; Group D: n = 27; high EOA risk group: n= 72; low EOA risk group: n = 27; high NDD risk group: n= 66; low NDD risk group: n = 21).
